# Effects of exercise on body mass index and waist circumference of individuals with intellectual and developmental disabilities: a systematic review with meta-analysis

**DOI:** 10.3389/fphys.2023.1236379

**Published:** 2023-08-02

**Authors:** Miguel Jacinto, Diogo Monteiro, Raul Antunes, José Pedro Ferreira, Rui Matos, Maria João Campos

**Affiliations:** ^1^ Faculty of Sport Sciences and Physical Education, University of Coimbra, Coimbra, Portugal; ^2^ ESECS—Polytechnic of Leiria, Leiria, Portugal; ^3^ Life Quality Research Centre (CIEQV), Leiria, Portugal; ^4^ Research Center in Sport Sciences, Health Sciences and Human Development (CIDESD), Vila Real, Portugal; ^5^ Center for Innovative Care and Health Technology (ciTechCare), Polytechnic of Leiria, Leiria, Portugal; ^6^ Research Center for Sport and Physical Activity (CIDAF), Coimbra, Portugal

**Keywords:** anthropometric assessment, body mass index intellectual disability, obesity, overweight, waist circumference

## Abstract

**Introduction/Methods:** This systematic review with meta-analysis aims to assess the magnitude of the effects of physical exercise programs on body mass index (BMI) and waist circumference (WC) of individuals with Intellectual and Developmental Disabilities (IDD), metabolic and cardiovascular health markers.

**Results:** Considering the eligibility criteria, a final sample of nine articles was obtained. For BMI, the *Z*-value obtained to test the null hypothesis (difference between means is zero), showed a *Z* = −2.176 and *p* = 0.03. The highest magnitude of the effect was from the intervention with combined training (difference in means: −0.399), with a value of *Z* = −1.815 and *p* = 0.07. For WC, the *Z*-value is zero, showing a *Z* = −3.306 and *p* = 0.001. The highest magnitude of the effect was from the intervention with continuous cardiorespiratory training of −0.786, with a value of *Z* = −2.793 and *p* = 0.005.

**Discussion:** Physical exercise prevents increases in BMI and WC in individuals with IDD. Aerobic training seems to be more effective in promoting WC and combined training in BMI.

**Systematic Review Registration**: [PROSPERO], identifier [CRD42021255316].

## 1 Introduction

Obesity is a major public health problem due to its growing prevalence, as it increases the risk of developing various diseases such as cardiovascular or metabolic diseases (de Winter et al., 2009; Vancampfort et al., 2020), increasing mortality in earlier ages when compared to the general population (Hosking et al., 2016). Excessive adiposity results from an imbalance between energy intake and expenditure.

Body mass index (BMI) and abdominal adiposity assessed using waist circumference (WC), body composition and anthropometric variables, are essential markers to assess overweight and obesity and are associated to metabolic disease and QoL (Klein et al., 2007; Kobo et al., 2019). This measure are non-evasive methods widely used in individual with Intellectual and Developmental Disabilities (IDD) to measure nutritional status (Temple et al., 2010; Waninge et al., 2010) and individuals with IDD are more likely to be overweight or obese compared to the general population (Zwierzchowska et al., 2021).

A systematic review with meta-analysis carried out by Maïano and collaborators (Maïano et al., 2016) showed that children and adolescents with IDD were 1.54 and 1.89 times more likely to be overweight and obese, when compared to the population without disability. These results are transversal to all age groups, from children (Wang et al., 2018), to adolescents (Krause et al., 2016) and adults (de Winter et al., 2012). Several factors may influence this prevalence, such as: 1) being female (de Winter et al., 2012); 2) advancing in age (Ranjan et al., 2018); 3) having DS (Krause et al., 2016); 4) having a degree of mild or moderate disability (Ranjan et al., 2018); 5) genetic factors (Wang et al., 2018). Other additional factors such as socioeconomic level, perceptions and attitudes towards physical activity, health problems and other characteristics of the disability itself (McGillivray et al., 2013), may also play a determinant role in this prevalence.

Considering the BMI variable, Temple and collaborators (Temple et al., 2014), when evaluating 11,643 individuals with IDD, verified that 5.5% of the sample was underweight, 36.1% in the normal range, 24.7% overweight, and 32.1% obese. Concluded that levels of overweight and obesity were high. Likewise, Foley and collaborators (Foley et al., 2017), evaluating 4,174 individuals with IDD, he also found that 32% were overweight and 11% were obese. At the same time, 21% of the participants were above the cut-off for abdominal obesity.

High values of BMI and WC, show a high prevalence of overweight and obesity in individuals with IDD. These values are associated with high risk metabolic and cardiovascular disease, excessive health costs (Vohra et al., 2017; Wyszyńska et al., 2017) and increased risk of incidence and mortality (Parra-Soto et al., 2021). On the other hand, BMI and WC are recommended by ACSM (American College of Sports Medicine, 2021) as two possible measures of anthropometric and body composition for individuals with IDD.

The global impact of physical activity and physical exercise on BMI and WC in people with IDD is not known, nor is the most effective type of exercise training for promoting these variables. International guidelines recommend by WHO (World Health Organization, 2020) and ACSM (American College of Sports Medicine, 2021) identify physical activity and exercise as important tools to improve daily life and wellbeing with a positive impact in different age groups (Kim et al., 2019). For these people, the variable mentioned, when practiced regularly, seem to be associated with improvements not only in physical fitness but also in reducing the risk of the appearance of metabolic and cardiovascular disease, reducing health costs and promoting their QoL (Pestana et al., 2018; Jacinto et al., 2021).

Since all of this work is based on the Guidelines for Exercise Testing and Prescription for individual with IDD (American College of Sports Medicine, 2021), we consider aerobic, resistance and flexibility training. According to ACSM (American College of Sports Medicine, 2021) aerobic exercise is the ability of the circulatory and respiratory system to supply oxygen during sustained physical activity, resistance training is the capacity of muscle to exert force and flexibility is the range of motion available at a joint.

The main purpose of the present systematic review with meta-analysis is to measure the magnitude of effects of different types of physical exercise on BMI and WC, metabolic and cardiovascular health parameter, in individual with IDD aiming to provide relevant information to sport sciences and health sciences professionals when planning, implementing and monitoring exercise intervention programs in people with IDD.

## 2 Materials and methods

The present systematic review with meta-analysis followed the guidelines defined in the original checklist of Preferred Reporting Items for Systematic Reviews and Meta-Analyses—PRISMA (Page et al., 2021). The protocol has been registered at the PROSPERO International Propective Regiter of Sytematic Review, with a number 2021: CRD42021255316.

The PICOS strategy (Methley et al., 2014; Nang et al., 2015) was used to ensure rigor defining of the research question, in which: 1) “P” corresponded to participants with IDD of any age, regardless of ethnicity or gender; 2) “I” corresponded to any physical exercise program implemented in the population with IDD (DS included), regardless of the intervention time, according to ACSM (American College of Sports Medicine, 2021); 3) “C” (Comparison) corresponded to the comparison between the CG versus the; 4) “O” corresponded to BMI and WC as the first or second variable in focus; 5) “S” (Study Design) corresponded to randomized controlled clinical trials (RCT).

### 2.1 Data sources

The search was conducted in the English language, in the following electronic databases: PubMed (title and abstract), Web of Science, and Scopus (title, abstract and key words), accessed between February 2021 and December 2022, using the advanced search option, with randomized exercise intervention studies. The search has been updated until the 10th of December. The search strategy combined Key Medical Subject Heading and indexed search descriptors to refine the data, following the recommendation from the Cochrane Handbook for Systematic Review of Intervention (Higgins and Altman, 2008), as shown in Table 1.

**TABLE 1:** Research Content.

Research content
(“intellectual disability” OR “intellectual disabilities” OR “mental retardation” OR “Down Syndrome” OR “Intellectual Developmental Disorder” OR “Intellectual Developmental Disabilities” OR “Intellectual Developmental Disability”) AND (“exercise” OR “training”) AND (“body mass index” OR “waist circumference”).

### 2.2 Eligibility criteria and studies selection

To be included in the present systematic review with meta-analysis, studies must meet the following inclusion criteria: 1) RCT studies with exercise intervention (with intervention group and control group), with any prescription in terms of intensities and duration, according to ACSM guidelines (American College of Sports Medicine, 2021); 2) All participants mut have an IDD diagnosis, whatever the degrees, including other subgroups with IDD (diagnosis by Wechsler Adult Intelligence Scale—Fourth Edition (Wechsler, 2008) or The Wechsler Intelligence Scale for Children—Fifth Edition Integrated (Raiford, 2018); 3) Participants with IDD of any age, gender, race or ethnicity, regarding ACSM (American College of Sports Medicine, 2021); 4) Studies focusing on aerobic, neuromuscular, flexibility or combined capacity (training that combines more than one physical capacity, e.g., strength and aerobic capacity), which recommended by ACSM (American College of Sports Medicine, 2021), Figure 1 shows research content. In turn, all studies with the following characteristics were excluded: 1) Studies published in a language other than English; 2) Studies that do no describe the intervention protocol; 3) Studies with participants with another type of disability or other associated pathologies; 4) Studies in which the intervention is multidimensional (studies involving exercise and nutrition, exercise and health education sessions); 5) Studies that do not show anthropometric data (BMI and WC); 6) Studies that the intervention protocol is through virtual reality (institution where we want to replicate the protocol does not have access to this material, as well as other institutions where most of this population usually spends their day); sports programs. All studies that did not meet the initial selection criteria and did not report results adequately (mean, standard deviation and sample size) or if the respective authors did not reply to our inquiries sent by email, were excluded. Finally, articles presented in abstracts, letters to the editor, systematic reviews, study protocols, and book chapters were excluded.

**FIGURE 1:**
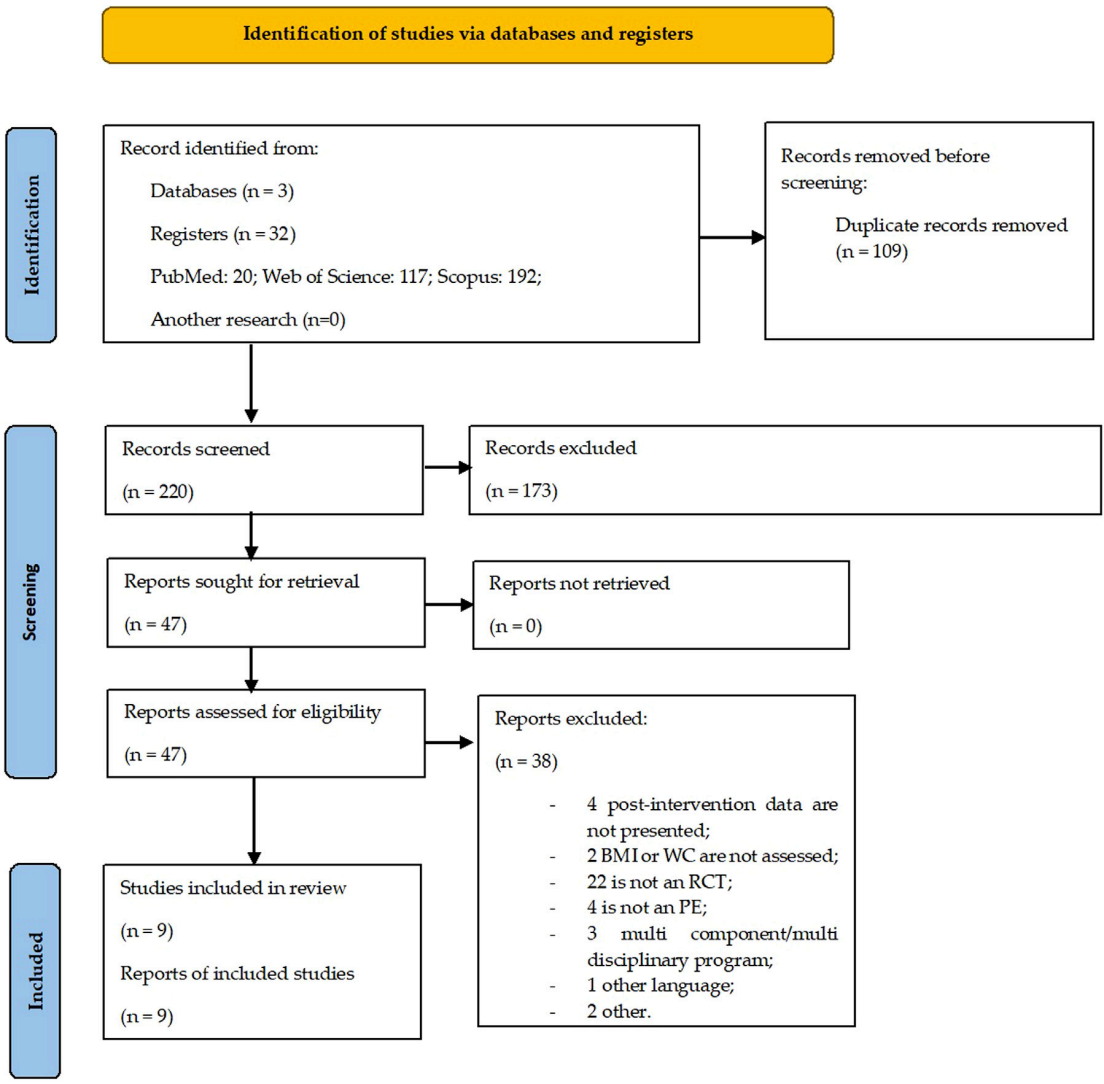
PRISMA flow diagram.

### 2.3 Data extraction

Studies were imported into EndNote X7 software, and duplicates were removed. The study selection procedure was carried out in phases. In the first phase, the search for potentially relevant studies was carried out with the participation of two independent reviewers, based on the titles and the abstract. These studies would proceed to the next evaluation phase in case of doubt following. In the second phase, the studies from the previous stage were reviewed by the same independent reviewers based on the application of the previously defined eligibility criteria. In case of doubt or disagreement regarding the inclusion of a study, this was solved through a third reviewer’s opinion playing the mediator’s role and whose decision was used as a tiebreaker. Finally, the first two reviewers involved in the selection of the studies participated independently in the analysis of the studies extracting all relevant information and characteristics, namely, the author’s name, year and country where it was carried out, objective, participants, instruments/techniques, duration/frequency, and results. In this phase, discrepancies about the extracted data were resolved by consensus among reviewers.

### 2.4 Quality assessment of studies

The PEDro Scale from the Physiotherapy Evidence Database, was used (Maher et al., 2003) to assess the quality of each study. The scale consists of 11 items, which analyse the different characteristics of each study, one of which is not counted (item 1) and the two others are not applicable in the field of sports science (items 5 and 6). The results obtained by both were compared and discussed so that there was a consensus. When there was no consensus, a third researcher was invited to collaborate.

### 2.5 Statistical analysis

Meta-analysis was performed using Comprehensive Meta-analysis Version 3.0 statistical software. The standardised difference in means was calculated based on information on pre and post-intervention means, number of participants, and standard deviation, using the randomized effects model to measure the effect size, the confidence interval (CI) of 95%, the magnitude of effects and level of statistical significance (*p* < 0,05). Favours A corresponds to, EG and Favour B correspond to CG. Heterogeneity was measured using chi-square, Cochran’s *Q* statistic, Higgin *I* squared (*I* ^*2*^), and Tau square tests (*T* ^*2*^). The Q statistic was used to test the null hypothesis, according to which all studies under analysis share a typical magnitude of effects. If all studies share the same effect-size, the expected values of *Q* would be equal to the number of degrees of freedom (N-1). *I* ^*2*^, which represents the percentage of variance attributed to the heterogeneity of the study, ranged from low (25%) to high (50%), with 50% being considered moderate (Batterham and Hopkins, 2006). *T* ^*2*^ is the variance of the true effect dimensions (in log units) between studies (Higgins et al., 2003), assuming that T^2^ > 1 suggests the presence of substantial heterogeneity. The homogeneity was verified by visualizing the asymmetry of the funnel-shaped scatter plot (Egger et al., 1997), considering that there was no publication bias when the graph had an inverted funnel (Higgins and Altman, 2008). Since the funnel-shaped scatter plot interpretation is sometimes subjective, the Egger test was used to check for publication bias (Rosenblad, 2009). Four meta-analyses were carried out, two to investigate the impact of exercise on the BMI and WC and another two to find out which type of training is most effective in provoking such adaptations.

## 3 Results

### 3.1 Data search

**FIGURE 2:**
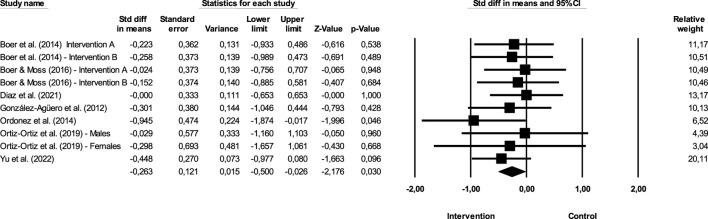
Difference of means of effect size comparing pre versus post intervention (BMI).

With the search carried out in different databases PubMed, Web of Science, and Scopus) 329 studies were identified. Subsequently, after eliminating the duplicate studies and reading the titles and abstracts, 47 studies with potential relevance to the study were identified. Considering the eligibility criteria previously defined for this systematic review with meta-analysis, from the complete reading of the articles, a sample of nine studies was constituted for their full analysis (Figure 2).

### 3.2 Characteristics of the studies

Details of the 9 studies included in the systematic review and assessed for quantitative analysis are presented in Table 2.

**TABLE 2:** Characteristics of the 9 selected studies.

Author	Aims	Participants	Assessment tools	Exercise program	Results	Quality score
Boer et al. (2014)	Effects of sprint interval training on metabolic and physical fitness.	N = 46	BMI (kg/m2); WC (tape: level of the umbilicus with the 92 subject in a standing position after normal expiration).	15 weeks; 2 x week; 40 min/session	BMI (pre vs. post) Intervention A: 28.4 ± 4.7 vs. 27.7 ± 4.7; Intervention B: 27.5 ± 2.7 vs. 26.9 ± 3.1; Control: 26.9 ± 3.2 vs. 26.9 ± 2.9.	5/8
		17 ± 3 years; IDD (fragile X syndrome, fetal alcohol syndrome, Prader–Willi and others)		Intervention A - Interval training: a sprint interval block (10 min), continuous aerobic exercise (10 min), and another sprint interval block (10 min); each sprint interval block consisted of 10 sprint bouts (>100 r/min) of 15s at a resistance matching with the VTR, alternated with 45s relative rest (50 r/min at VTR); 100%–110% of VTR	WC (pre vs. post) Intervention A: 95.8 ± 13.1 vs. 91.5 ± 13.1; Intervention B: 95.9 ± 9.6 vs. 93.4 ± 9.6; Control: 95 ± 8.8 vs. 95.9 ± 8.2.	
		Randomized groups: interval training—Intervention A (N = 17); continuous aerobic training—Intervention B (N = 15); control (N = 14).		Intervention B—Continuous aerobic training: cycling (10 min), walking/running (10 min), stepping (10 min); 100%–110% of VTR.		
Boer and Moss (2016)	Determine the effect of continuous aerobic training vs. interval training on several parameters.	N = 42; 33.8 ± 8.6 years; DS	Body weight; Height; WC (tape: measured at the umbilicus with the participant standing upright after a normal expiration).	12 weeks; 3 x week; 30 min/session	BMI (pre vs. post) Intervention A: 30.6 ± 6.1 vs. 30.2 ± 6.3; Intervention B: 29.3 ± 4 vs. 28.5 ± 4; Control: 31.2 ± 4.6 vs. 30.9 ± 4.2.	7/8
		Randomized groups: continuous aerobic training—Intervention A (N = 13), interval training—Intervention B (N = 13); control (N = 16).		Intervention A—Continuous aerobic training: cycling or walking at an intensity of 70%–80% of VO_2max_	WC (pre vs. post) Intervention A: 95 ± 11.1 vs. 93.7 ± 11.9; Intervention B: 94.2 ± 8.1 vs. 93.8 ± 8; Control: 99.4 ± 10.9 vs. 98 ± 10.6.	
				Intervention B—Interval training: 10–30 s all out sprints with the 90 s (1:3 work-rest ratio) of low cadence, low intensity walking or cycling.		
Diaz et al. (2021)	Analyze the impact of circuit RT on markers of muscle damage.	N = 36; mean age 28.1 ± 3.3 years; DS	BMI (kg/m2); WC (tape: between the costal edge and the iliac crest).	12 weeks; 3 x week; Duration of session: NA	BMI (pre vs. post): EG: 31.4 ± 5.7 vs. 31.6 ± 6; CG: 30.8 ± 5.2 vs. 31 ± 5.5.	7/8
		Randomized groups: exercise (N = 18); control (N = 18).		Resistance circuit training: 40%–65% 8RM; 2 sets; 6 to 10 reps; 90s rest between stations; Exer: arm curl (elbow flexion), triceps extension (elbow extension), leg extension, seated row, leg curl (knee flexion), and leg press (combined hip and knee extension)	WC (pre vs. post): EG: 91.4 ± 12.8 vs. 90.8 ± 13.4; Control group: 88.9 ± 13.3 vs. 89 ± 13.4.	
González-Agüero et al. (2012)	Effect of training on bone mass.	N = 28; 10–19 years; DS	Height; Weight; BMI (kg/m^2^)	21 weeks; 2 x week; 25 min/session	BMI (pre vs. post): EG: 19.6 ± 2.7 vs. 20.2 ± 2.6; CG: 22.4 ± 3.4 vs. 22.3 ± 3.2.	5/8
		Randomized groups: exercise (N = 14); control (N = 14).		1 or 2 rotations in a four-stage circuit: jumps, press-ups on the wall, fitness bands and medicine balls.		
Ordonez et al. (2014)	Influence of aerobic training on pro-inflammatory cytokines and acute phase proteins.	N = 20; 8–30 years; DS	Height; Body weight; BMI (kg/m2); WC (tape: between the costal edge and the iliac crest).	10 weeks; 3 x week; 45–65 min/session.	BMI (pre vs. post): EG: 30.2 ± 0.9 vs. 29.8 ± 0.7; CG: 30.7 ± 0.8 vs. 30.9 ± 0.8.	5/8
		Randomized groups: exercise (N = 11); control (N = 9).		Aerobic training: 30–40 min treadmill exercise at 55%–65% of peak heart rate.	WC (pre vs. post): EG: 94.7 ± 3.3 vs. 91.5 ± 3.1; CG: 93.5 ± 3.1 vs. 93.7 ± 3.2.	
Ortiz-Ortiz et al. (2019)	Effect of a exercise on body composition and isometric strength.	N = 22; 8–16 years; DS	Height; Weight; BMI (kg/m^2^).	16 weeks; 5 x week; 55 min/session	BMI (pre vs. post) Males: 21.1 ± 1.8 vs. 19.7 ± 1.8; Females: 23.2 ± 2.9 vs. 21.5 ± 3; CG: Males: 23.3 ± 6.3 vs. 21.8 ± 5.9; CG Females: 23.3 ± 1 vs. 22.2 ± 0.9	7/8
		Randomized groups: exercise (N = 13); control (N = 9).		Strength training: circuit and “tabata” exercises; Different materials were used, such as weight discs, tension ropes, dumbbells, medicine balls, and handgrips; Exer: biceps curl, triceps extension, chest press, and handgrip with different degrees of tension.		
Rosety-Rodriguez et al. (2014)	Determine for how long the anti-inflammatory effect induced by aerobic training.	N = 20; 18–30 years; DS	WC (tape: do not mention the procedures).	10 weeks; 3 x week; 60 min/session.	WC (pre vs. post): EG: 94.7 ± 3.3 vs. 91.5 ± 3.1; CG: 93.5 ± 3.1 vs. 93.7 ± 3.2.	4/8
		Randomized groups: exercise (N = 11); control (N = 9).		Aerobic training: 30–40 min—treadmill; 55%–65% of peak heart rate.		
Shields and Taylor (2015)	Feasibility of a physical activity program.	N = 16; 18–35 years; DS	WC (tape: do not mention the procedures).	8 weeks; 3 x week; 150 min of moderate intensity PA per week	WC (pre vs. post): EG: 95.6 ± 17.2 vs. 90.1 ± 12.1; CG: 89.3 ± 8.8 vs. 94.1 ± 7.4.	8/8
		Randomized groups: exercise (N = 8); control (N = 8).		Aerobic training: walking.		
Yu et al. (2022)	Effectiveness school-based adapted physical activity.	N = 61; 12–18 years; IDD	BMI: (kg/m2); WC (tape: midway point between the lowest rib margin and the top of the iliac crest at the end of a gentle ventilatory expiration).	36 weeks; 2 x week; 45–60 min/session	BMI (pre vs. post): EG: 28.16 ± 3.69 vs. 27.5 ± 3.97; CG: 27.37 ± 3.99 vs. 28.05 ± 3.75.	6/8
		Randomized groups: EG (N = 39); CG (N = 22).		Aerobic training: 30%–60% peak heart rate—walk/run	WC (pre vs. post): EG 93.55 ± 10.11 vs. 91.54 ± 11.1; CG: 90.51 ± 11.72 vs. 92.27 ± 9.26.	
				Strength training: jumping jacks, high kness, sit-ups; 10–30s/sets; 4 sets; 1 min to 40 s break between sets.		

Note: CG, control group; EG, exercise group; DS, down syndrome; Exer, Exercise/s; Min, minutes; N, participants; Rep, repetitions; RM, maximum repetition; s, Seconds; SD, standard deviation; VO_2max_, maximum oxygen consumption; VTR, ventilatory threshold; *, Only analysed the exercise and control group.

### 3.3 Quality of the information

Rosety-Rodriguez and collaborators (Rosety-Rodriguez et al., 2014) were the studies that obtained the lowest quality score (4 points—40%), and the studies with the best scores had 8 points (80%) Shields and Taylor (Shields and Taylor, 2015), showing a good quality of the methodological procedures.

### 3.4 Participants

The total number of participants included in the different studies was 291, 172 in experimental groups and 119 in CG. The studies included different types of IDD, whether it is DS, autism, or others. In Boer et al. study (Boer et al., 2014), participants were attending 40 secondary school at two Belgian special education school. In Boer and Moss study (Boer and Moss, 2016), participants were recruited from three care centres for persons with IDD. Participants in the Diaz and collaborators study (Diaz et al., 2021) and Ordonez and collaborators (Ordonez et al., 2014) were recruited via community support groups for people with IDD. Also, González-Agüero and collaborators (González-Agüero et al., 2012) recruited participants from different schools and institutions. Ortiz-Ortiz (Boer et al., 2014) and Rosety-Rodriguez and collaborators (Rosety-Rodriguez et al., 2014) does not mention where and how participants were recruited. Shields and Taylor (Shields and Taylor, 2015) recruited participants by contacting family members who were interested and Yu and collaborators (Ortiz-Ortiz et al., 2019) recruited participants from six special schools for adolescents with mild/moderate IDD.

### 3.5 Duration

The exercise intervention programs ranged from 8 to 36 weeks, however is more prevalent a prescription of 10–12 weeks (Ordonez et al., 2014; Rosety-Rodriguez et al., 2014; Boer and Moss, 2016; Diaz et al., 2021), i.e., short duration programs. The two combined exercise programs included in this systematic review lasted for 21–36 weeks (González-Agüero et al., 2012; Yu et al., 2022), one of neuromuscular capacity exercise programs lasted 16 weeks (Ortiz-Ortiz et al., 2019) and other 12 weeks (Diaz et al., 2021) and the five aerobic exercise programs lasted from 8 to 15 weeks, with half being implemented over 10 weeks (Boer et al., 2014; Ordonez et al., 2014; Rosety-Rodriguez et al., 2014; Shields and Taylor, 2015; Boer and Moss, 2016).

The frequency varied between 2 and 5 times per week, with most studies implementing 3 times per week (Ordonez et al., 2014; Rosety-Rodriguez et al., 2014; Shields and Taylor, 2015; Boer and Moss, 2016; Diaz et al., 2021). The two combined exercise programs included in this systematic review with meta-analysis have a frequency of 2 times per week (González-Agüero et al., 2012; Yu et al., 2022). Regarding neuromuscular capacity, one of the exercise programs have a frequency of 5 times per week (Ortiz-Ortiz et al., 2019) and other 3 times per week (Diaz et al., 2021). Finally, the 5 aerobic exercise programs have a frequency of 2 and 3 times per week, with the majority implemented 3 times per week (Boer et al., 2014; Ordonez et al., 2014; Rosety-Rodriguez et al., 2014; Shields and Taylor, 2015; Boer and Moss, 2016). Regarding the duration of the exercise intervention session, sessions varied between 25 and 65 min including a brief warm-up and a return to calm period. The duration of the training session in the two combined exercise programs varied from 25 to 60 min (González-Agüero et al., 2012; Yu et al., 2022), one of the exercise programs for neuromuscular capacity were implemented for 55 min (Ortiz-Ortiz et al., 2019), with the other one not showing the session duration (Diaz et al., 2021), and from the five aerobic exercise programs four lasted from 30 to 60 min (Boer et al., 2014; Ordonez et al., 2014; Rosety-Rodriguez et al., 2014; Boer and Moss, 2016). One of the studies did not mention the duration of the training session, but mentions the weekly volume, namely, 150 min per week (Shields and Taylor, 2015).

### 3.6 Type of exercise program

Concerning aerobic training, different intensities were reported following the global recommendations/guidelines presented of the ACSM (American College of Sports Medicine, 2021) for efforts within the interval of 60%–85% of maximum heart rate (HRmax).

Some studies used an intensity of 40%–65% HRmax (Ordonez et al., 2014; Rosety-Rodriguez et al., 2014; Diaz et al., 2021), others used 100%–110% of the ventilatory threshold (Boer et al., 2014) while others reported a 70%–80% maximum oxygen consumption (VO2max) (Boer and Moss, 2016) intensity value, with gradual increments throughout the intervention. These studies use different equipment such as stationary cycling, treadmills, or other materials such as steps or walking/running.

Interval training programs demonstrate a reduced volume compared to continuo training and used periods of 10 s of maximum speed, followed by 90 s of rest (Boer and Moss, 2016) or 15 s of full speed followed by 45 s of rest (Boer et al., 2014) using cycle ergometers or simple walks/runs.

All the exercise programs focused on neuromuscular capacity used a training circuit with different materials. The study by Diaz and collaborators (Diaz et al., 2021) worked at loads of 40%–65% of 8 repetition maximum (RM). One of the combined training programs is based time set (10–30 s per set; 4 sets) and aerobic intensity interval with a HRmax between 30% and 60% (Yu et al., 2022), and a second one is a four-stage circuit based on training with body weight, fitness bands and medicine balls (González-Agüero et al., 2012).

### 3.7 Results of intervention on BMI

Figure 2 show the impact of exercise on BMI.

The sum of the effects is −0.263, which means that individuals in the, EG are 0.263 times more likely to report decreases when compared to the CG when the inclusion and exclusion criteria previously described in the study are met. The CI for the difference in means is from −0.5 to −0.026, which means that the gross disparity in means, in the universe of studies, may fall somewhere in this interval. On the other hand, this range does not include the difference of zero, which means that the true difference in means is different from zero. The *Z* values obtained to test the null hypothesis, according to which the difference between means is zero, showed a *Z* = −2.176, with the corresponding value of *p* = 0.03. The obtained value of *Q* is 3.856 with 9 degrees of freedom and with a *p* ≥ 0.05. We cannot reject the null hypothesis that the true magnitude of effects is the same across studies, and we can say that the true extent of effects does not varies from study to study. In the present meta-analysis, the *I* ^2^ value obtained is 0, which means that the variance in the observed effects reflect 0% the variance in the true results. On the other hand, *T* ^2^ corresponds to the variance of the true magnitude of the impact (true effect sizes) between studies that, in the present study, have a value of 0, as well as the value of *T*, concerning the deviation pattern of the true magnitude of the effects.

**FIGURE 3:**
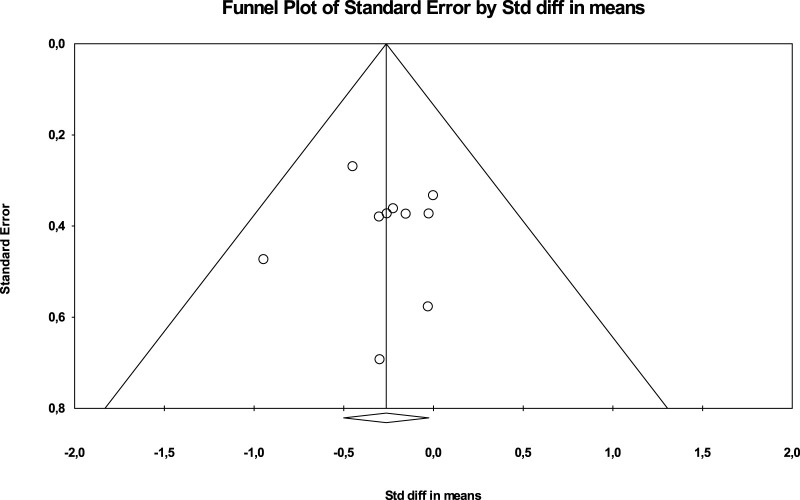
Scatterplot in funnel format for verification of publication bias (BMI).

Figure 3 show the publication bias.

**FIGURE 4:**
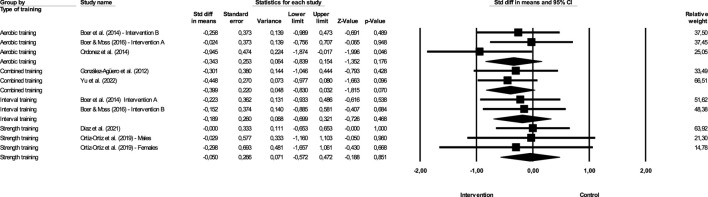
Difference in effect size means comparing different exercise (BMI).

In addition, the Egger test was carried out, which proposes to test the null hypothesis according to which the intercept is equal to zero in the population. For Figure 4, the intercept is −0.03655, 95% CI (−2.20324, −2.13014), with *t* = 0.0389, degrees of freedom = 8 The recommended value of *p* (2-tailed) is 0.96. There is no statistical evidence for the existence of publication bias.

#### 3.7.1 Most effective type of training to improve BMI

Figure 4 show the impact of different exercise on BMI.

Continuous aerobic training—The magnitude of the effect of the intervention with aerobic training was −0.343, with a value of *Z* = −1.352 and *p* = 0.176. Combined training—The effect of the intervention with interval training was −0.399, with a value of *Z* = −1.815 and *p* = 0.07. Interval training—The effect of the intervention with interval training was −0.189, with a value of *Z* = −0.726 and *p* = 0.468. Strength training—The magnitude of the effect of the intervention with strength training was −0.05, with a value of *Z* = −0.188 and *p* = 0.851. In this case, *Q* = 1.202 with 3 degrees of freedom and *p* > 0.05. We can accept the null hypothesis that the actual effect size is the same in all studies. However, the study that shows the greatest effectiveness is combined training, which has a higher magnitude of effect, although the difference between the effects of different studies is not significant.

### 3.8 Results of intervention on WC

**FIGURE 5:**
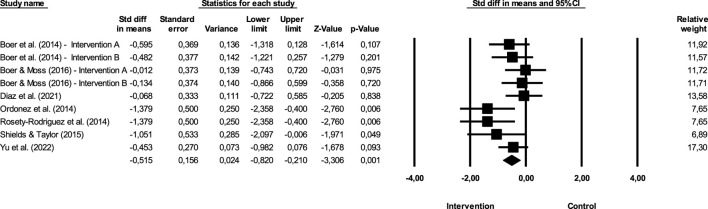
Difference in mean effect size comparing pre versus post intervention (WC).

Figure 5 show the impact of exercise on WC.

The sum of the effects is −0.515, which means that individuals in the, EG are 0.515 times more likely to report decreases when compared to the CG when the inclusion and exclusion criteria previously described in the study are met. The CI for the difference in means is from −0.82 to −0.21, which means that the gross disparity in means, in the universe of studies, may fall somewhere in this interval. On the other hand, this range does not include the difference of zero, which means that the true difference in means is different from zero. The *Z* values obtained to test the null hypothesis, according to which the difference between means is zero, showed a *Z* = −3.306, with the corresponding value of *p* = 0.001. The obtained value of *Q* is 11.683 with 8 degrees of freedom and with a *p* = 0.166. We cannot reject the null hypothesis that the true magnitude of effects is the same across studies, and we cannot say that the true extent of effects varies from study to study. In the present meta-analysis, the *I* ^*2*^ value obtained is 31.526, which means that the variance in the observed effects does not reflect the variance in the true results (just reflect 31%). On the other hand, *T* ^*2*^ corresponds to the variance of the true magnitude of the impact (true effect sizes) between studies that, in the present study, have a value of 0.067, as well as the value of *T*, concerning the deviation pattern of the true magnitude of the effects.

**FIGURE 6:**
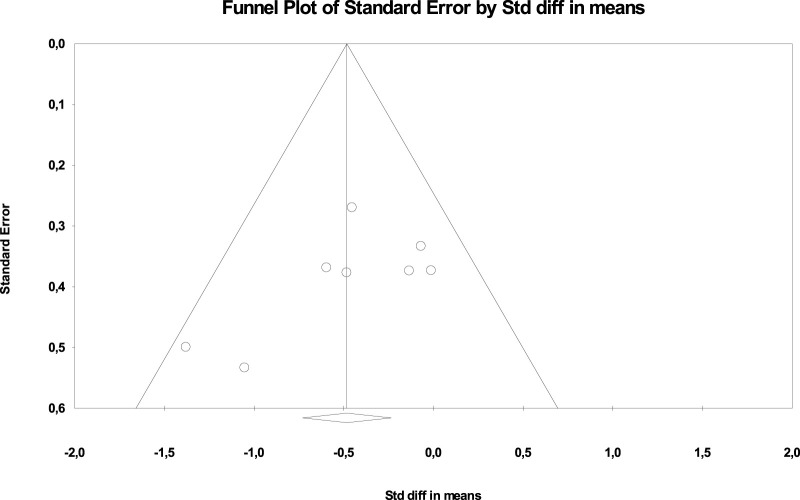
Scatterplot in funnel format for verification of publication bias (WC).

Figure 6 show the publication bias.

**FIGURE 7:**
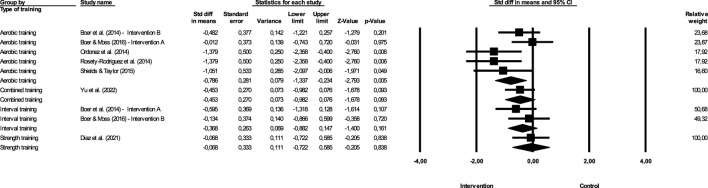
Difference in mean Effect size comparing different exercise (WC).

In addition, the Egger test was carried out, which proposes to test the null hypothesis according to which the intercept is equal to zero in the population. For Figure 7, the intercept is −3.7287, 95% CI (−7.40536, −0.04837), with *t* = 2.39572, degrees of freedom = 7. The recommended value of *p* (2-tailed) is 0.04777. There is statistical evidence of the existence of publication bias.

#### 3.8.1 Most effective type of training to improve WC

Figure 7 show the impact of different exercise on WC.

Continuous aerobic training—The effect of the intervention with continuous aerobic training was −0.786, with a value of *Z* = −2.793 and *p* = 0.005. Combined training—The effect of the intervention with combined training was −0.453, with a value of *Z* = −1.678 and *p* = 0.093. Interval training—The effect of the intervention with interval training was −0.368, with a value of *Z* = −1.4 and *p* = 0.161. Strength training—The magnitude of the effect of the intervention with strength training was −0.068, with a value of *Z* = −0.205 and *p* = 0.838. In this case, *Q* = 2.831 with 3 degree of freedom and *p* > 0.05, so we can accept the null hypothesis that the actual effect size is the same in all studies. However, the study that shows the greatest effectiveness is continuous aerobic training, which has a higher magnitude of effect, although the difference between the effects of different studies is not significant.

## 4 Discussion

This systematic review with meta-analysis aimed to assess the magnitude of the effects of different types of exercise programs on BMI and WC, variables related to metabolic and cardiovascular health of individuals with IDD.

The results of exercise programs are varied, depending on the objectives and the assessment tools/techniques. The fact that the present systematic review encompassed people with IDD of varying degrees and diagnoses (DS, autism, or others) may have influenced our results, because subgroups may have different responses to exercise ACSM (American College of Sports Medicine, 2021). However, these different responses to exercise still need further study to determine the optimal exercise intensities and modes for the population. However, taking into account the purposes of this systematic review with meta-analysis, we found that all studies that assess the BMI (Boer et al., 2014; Ordonez et al., 2014; Boer and Moss, 2016; Ortiz-Ortiz et al., 2019; Yu et al., 2022) and WC (Boer et al., 2014; Ordonez et al., 2014; Rosety-Rodriguez et al., 2014; Shields and Taylor, 2015; Boer and Moss, 2016; Yu et al., 2022) had a decrease in the values of these same variables through the implementation of exercise programs, except studies by González-Agüero and collaborators (González-Agüero et al., 2012) and Diaz and collaborators (Diaz et al., 2021), where there were an increases in BMI. In the González-Agüero and collaborators (González-Agüero et al., 2012) study, it was natural to see an increase in BMI due to the aim of the study. On the other hand, this increase was beneficial due to the relatively low mean BMI values of the sample, according to the cut-off values, in contrast to most of the literature. In the Diaz and collaborators (Diaz et al., 2021) study, the increase in BMI values may be justified by increases in muscle mass.

All studies used the same paradigm, whereby individuals with IDD were randomly placed in the experimental group (with exercise) or the CG. There is a shortage of exercise programs with randomized controlled methodology that assesses the impact on BMI and WC, along with only the population with IDD. The results were reported regarding the improvement of the BMI or WC.

Exercise was different in the studies, also differing in the physical capacity for training (aerobic training, strength, and/or combined training). The most used training methodology is the continuous aerobic type (Boer et al., 2014; Ordonez et al., 2014; Rosety-Rodriguez et al., 2014; Shields and Taylor, 2015; Boer and Moss, 2016), with observing a reduced or null number of interventions focusing on other physical fitness components. Therefore, which presupposes that the results of this study should be taken with caution.

Considering the present systematic review with meta-analysis, exercise had superior effects in most studies. However, the differences were not significant in some studies. Thus, we can reject the null hypothesis that exercise does not affect the BMI or WC of individuals with IDD, on the other hand, exercise seems decreases BMI and WC values. This is the strength of the study, since previous research shows that exercise interventions did not promote BMI and WC of individuals with IDD (Harris et al., 2015), even multi-component weight management interventions, namely, inclusion of an energy deficit diet, physical activity, and behaviour change techniques, are effective (Harris et al., 2018) and that only exercise and diet interventions could promote the variables under study (Harris et al., 2018; Ptomey et al., 2018). Currently, more researchers interested in promoting the QoL of these individuals may be at the origin of the results of the present study (Schalock et al., 2002), since recommendations for the assessment and prescription of exercise in individuals with IDD are frequently published, adapted, from previously implemented studies (American College of Sports Medicine, 2021). This increased interest increases knowledge of effective strategy and methodologies for QoL improvement. Since individuals with disabilities usually have high levels of overweight and obesity, the results of this study highlight the importance of regular exercise practice by individuals with IDD, to prevent the increase in values such as BMI and WC and, consequently, prevent the onset of metabolic and cardiovascular diseases. On the other hand, a follow-up by the technical of exercise, in order to assess and prescribe exercise in a correct and adapted way should be considered (American College of Sports Medicine, 2021).

According to this systematic review with meta-analysis, combined training appears to be the most efficient method for the promotion of BMI and aerobic training for WC and, in turn, the metabolic health of individuals with IDD. The literature is not clear about the training methodology that best promotes the variables under study. For Skrypnik and collaborators (Skrypnik et al., 2015) there are no significant differences between the different methods. Aerobic training reduces fat mass but has little effect on maintaining fat free mass (Garrow and Summerbell, 1995), and some authors point out that it is effectively the best method to reduce body mass (Willis et al., 2012). However, strength training, which produces fat-free mass gain, also increases resting energy expenditure (Hunter et al., 2000). Exercise combined resistance and aerobic training showed to be a good alternative for increasing fat-free mass and reducing fat mass (Willis et al., 2012), with authors claiming that it is the best method for losing weight and fat mass and maintaining fat free mass (Ho et al., 2012).

This article investigates which type of intervention best promotes BMI and WC in individuals with IDD. However, the small number of articles included and heterogeneity of population and diagnosis in the meta-analysis and a higher prevalence of studies with continuous aerobic methodology may have limited the results. It is recommended to continue implementing exercise programs with different methods, focusing on physical abilities in isolation or combination, so that further studies can measure these results way more precisely and robustly. On the other hand, waist circumference, may be considered a limitation of the present study, despite its usefulness, low cost and wide availability in any clinical setting, due to measurement errors because of its lack of reproducibility (Bouchard, 2007). Several studies are recommending the use of imaging techniques as they are more accurate and reproducible, however, they are also more expensive and complex (El Ghoch et al., 2012). At the same time, we recommend that future studies investigate the impact of a multidisciplinary intervention on these variables. Seeing if it can have more impact than exercise alone. We also recommend that future interventions are aimed at reducing energy intake and not just energy expenditure through the exercise.

## 5 Conclusion

Based on the results of the systematic review with meta-analysis, we can affirm that exercise programs prevent BMI and WC increments of individuals with IDD. Although without significant results, combined training looks to be more effective in promoting BMI and continuous aerobic training for WC since it had a greater effect size. The interest of various stakeholders in studying the QoL of individuals with IDD has increased, and the results of this systematic review with meta-analysis should be considered when planning interventions with the focus populations, in the sense that exercise programs promote BMI and WC, which, in turn, is associated with metabolic and cardiovascular health. The practice of exercise, in addition to promoting physical capacity, reduces the risk of diseases, being an essential aspect for a better QoL in individuals with IDD.

## Data Availability

The original contributions presented in the study are included in the article/Supplementary Material, further inquiries can be directed to the corresponding author.
